# Serum zonulin as an index of glucose dysregulation in children and adolescents with overweight and obesity

**DOI:** 10.1111/ijpo.12946

**Published:** 2022-06-06

**Authors:** Francesca Olivieri, Alice Maguolo, Massimiliano Corradi, Chiara Zusi, Valentina Huber, Elena Fornari, Anita Morandi, Claudio Maffeis

**Affiliations:** ^1^ Pediatric Diabetes and Metabolic Disorders, Department of Surgical Sciences, Dentistry, Paediatrics and Gynaecology University of Verona Verona Italy

**Keywords:** children and adolescents, insulin resistance, intestinal permeability, lipopolysaccharide, obesity, type 2 diabetes mellitus, zonulin

## Abstract

Increased intestinal permeability has an important role in metabolic dysregulation. In this cross‐sectional study, we examined whether serum intestinal permeability marker zonulin and related pro‐inflammatory molecules were associated with the oral disposition index, a predictor for the development of type 2 diabetes, in a cohort of children and adolescents with overweight and obesity. Ninety‐two children and adolescents were recruited [Male: 43; 12.7 (2.35) years; BMI SDS: 2.7 (0.96)]. Anthropometric and clinical parameters, lipid profile, glucose metabolism and plasma levels of zonulin, lipopolysaccharide‐binding protein and Interleukin‐6 were measured. We found an association between oral disposition index and zonulin (β = −0.243; *p* = 0.019) and age (β = −0.307; *p* = 0.004), independent of sex and BMI SDS [R^2^ = 0.16; *p* = 0.005]. Our results show an association between serum zonulin concentration and oral disposition index supporting the hypothesis of increased intestinal permeability as a possible risk factor for glucose metabolism dysregulation in children and adolescents with obesity.

**Abbreviations**
ALT: alanine aminotransferase
BMI: body mass index
CVD: cardiovascular disease
HOMA‐IR: Homeostatic Mode Assessment for Insulin Resistance
IL‐6: Interleukin‐6
LBP: Lipopolysaccharide‐binding protein
LPS: lipopolysaccharide
MetS: metabolic syndrome
OGTT: oral glucose tolerance test
SDS: standard deviation score
T2DM: type 2 diabetes mellitus
TC: total cholesterol

## 1 BACKGROUND

Obesity is a major risk factor for type 2 diabetes mellitus (T2DM), dyslipidaemia, hypertension and cardiovascular disease.^1^ Having obesity in childhood increases the risk of developing T2DM fourfold. Moreover, insulin resistance accompanying obesity plays a key role in the development of diabetes and metabolic syndrome (MetS).^1^ A better understanding of the underlying causes of insulin resistance and early identification of children and adolescents with obesity most at risk of metabolic complications is crucial in clinical practice to implement targeted and early preventive strategies.^2^ Intestinal permeability has been proposed as one of the main factors involved in the development of insulin resistance.^3^ An increased intestinal permeability mediates an altered transit of substances and products, as pro‐inflammatory molecules, through the intestinal wall to the systemic circulation, resulting in an alteration of the insulin receptor signalling via multiple pathways.^3^ The presence of altered intestinal permeability is not easy to assess and, recently, serum zonulin, a protein produced by the intestinal epithelium that mediates the destruction of enterocytes' tight junctions, has been proposed as a readily available indicator.^4^, ^5^ The aim of this study was to determine whether zonulin and related pro‐inflammatory molecules are associated with the oral disposition index (oDI), as a risk factor for the development of T2DM, in a cohort of children and adolescents with overweight and obesity.

## 2 METHODS

Ninety‐two children and adolescents [Males: 43; mean age 12.7 (2.35) years; BMI SDS: 2.7 (0.96)] were enrolled at the Regional Center for Paediatric Diabetes, University Hospital, Verona (Italy). Inclusion criteria were: European ethnicity, overweight and obesity according to WHO sex and age‐specific BMI cut off.^6^ Exclusion criteria: genetic or endocrine causes of obesity, type 1 diabetes mellitus or other genetic forms of diabetes, malignancy, associated chronic diseases or chronic pharmacological therapies potentially affecting glucose metabolism, celiac disease or other diseases characterized by malabsorption. None of the children included was affected by T2DM. The protocol was approved by the Institutional Ethics Committee for Clinical Experimentation of Verona and Rovigo (CESC VR‐RO) (Italy). Written informed consent was obtained from the children and their parents.

At recruitment, anthropometric and physical evaluation was performed according to standard procedures and data on BMI SDS, waist circumference, Tanner pubertal stage, systolic and diastolic blood pressure (SBP, DBP), as previously described,^7^ were collected. Within 15 days from the recruitment visit, patients underwent fasting blood tests for measuring plasma glucose, serum insulin concentration, glycated haemoglobin (HbA1c), lipid profile and alanine aminotransferase (ALT).^7^ Participants underwent a standard 75 g oral glucose tolerance test (OGTT) after overnight fasting, and the following OGTT‐derived indexes were calculated: insulinogenic index (IGI) as [insulin_30’_ (mU/L) − insulin_0’_ (mU/L)]/[glucose_30’_ (mg/dl)‐glucose_0’_ (mg/dl)]; Matsuda index as 10 000/[(Glucose_0’_ (mg/dl) × Insulin_0’_ (mU/L)) × (mean OGTT glucose concentration (mg/dl)) × (mean OGTT insulin concentration (mU/L))]^1/2^ and oral disposition index (oDI) as marker of the adequacy of insulin bioavailability to the prevailing insulin sensitivity given by the formula (Matsuda index×IGI).^8^

Plasma levels of zonulin (ng/ml), lipopolysaccharide‐binding protein (LBP) (μg/ml), and Interleukin‐6 (IL‐6) (pg/ml) were measured by ELISA commercial kit (Immundiagnostic K5601, ABNOVA KA0448, and Invitrogen BMS 213HS, respectively). For serum zonulin kit (intra assay CV 6%, inter assay CV 8.3%), according to the manufacturer, a mean value of 34 ng/ml (±14 ng/ml) was declared as normal for healthy persons. The used antibody presents no cross‐reactivity against human haptoglobin while the limit of quantitation is 0.183 ng/ml. LBP directly binds lipopolysaccharide (LPS) that belongs exclusively to the Gram‐negative bacteria cell wall and is therefore an indicator of intestinal dysbiosis and inflammation, both causes of increased intestinal permeability. IL‐6, a proinflammatory cytokine, is an index of the systemic outcomes of elevated intestinal permeability and mediates dysregulating effects on lipid and glucose metabolism. Moreover, IL‐6 triggers the transcription of zonulin, promoting the establishment of a vicious cycle that induces increased permeability.^3^

Kolmogorov–Smirnov test was used to assess the normal distribution of variables. Skewed variables were natural log‐transformed to correct for non‐Gaussian distribution. The Student's *t*‐test and the Pearson's chi‐squared test were used to evaluate the differences in clinical and biochemical characteristics according to sex. Linear regression models, adjusted for age, sex and BMI SDS, were applied to test the independent associations between intestinal permeability indices and oDI. A *p*‐value <0.05 was considered as statistically significant. All analyses were performed using SPSS v.26.0 (SPSS, Chicago IL).

## 3 RESULTS

Ninety‐two children and adolescents with overweight and obesity [males = 43; mean age 12.7 (2.35); min‐max: 8,5–18 years) and a mean BMI SDS of 2.7 (0.96) were recruited. Anthropometric and metabolic characteristics of the study sample are shown in Table 1. We found a correlation between zonulin and oDI (Pearson ρ = −0.256, *p* value = 0.015). Linear regression analysis showed a significant association between BMI SDS and zonulin (β = 0.233; *p* = 0.030), LBP (β = 0.384; *p* < 0.001), and IL‐6 (β = 0.447=; *p* < 0.001), independent of age and sex. In Table 2, linear regression model revealed an association between oDI and zonulin (β = −0.243; *p* = 0.019) and age (β = −0.307; *p* = 0.004), independent of sex and BMI SDS [R^2^ = 0.16; *p* = 0.005]. These associations were independent of pubertal stage (Table 2).

**TABLE 1:** Clinical and anthropometric characteristics of the total sample and according to sex

	M (*n* = 43)	F (*n* = 49)	Total (*n* = 92)	*p*‐value
Age (years)	12.2 (2.2)	13.2 (2.4)	12.7 (2.35)	ns
Weight (kg)	74.8 (23.8)	82.8 (27.3)	79.0 (25.92)	ns
Height (cm)	157.6 (13.5)	158.2 (10.4)	157.9 (11.89)	ns
BMI (kg/m^2^)	29.4 (6.1)	32.4 (8.2)	31.0 (7.43)	ns
BMI‐SDS	2.7 (1.0)	2.8 (1.0)	2.7 (0.96)	ns
PAS (mmHg)	112.6 (14.2)	113.0 (10.4)	112.8 (12.2)	ns
PAD (mmHg)	69.5 (7.5)	67.4 (7.9)	68.4 (7.7)	ns
Puberty (n = 71)				<0.001
Prepubertal	7 (21.2%)	4 (10.5%)	11 (15.5%)	
Pubertal	24 (72.2%)	11 (28.9%)	35 (49.3%)	
Postpubertal	2 (11.6%)	23 (60.5%)	25 (35.2%)	
TG (mg/dl)	86.7 (33.3)	94.8 (45.8)	91.1 (40.6)	ns
TC (mg/dl)	148.6 (29.1)	151.5 (31.0)	150.2 (30.0)	ns
HDL‐c (mg/dl)	48.3 (8.8)	44.4 (10.2)	46.1 (9.7)	ns
LDL‐c (mg/dl)	83.0 (28.8)	88.2 (24.5)	85.9 (26.4)	ns
ALT (U/L)	30.9 (13.8)	25.8 (13.1)	28.0 (13.5)	ns
HbA1C (mmol/mol)	37.2 (3.4)	38.2 (4.7)	37.7 (4.1)	ns
Glycaemia (mg/dl)	95.4 (5.4)	94.4 (8.2)	94.9 (7.0)	ns
Glycaemia 120′ (mg/dl)	118.2 (20.0)	116.0 (26.1)	117.0 (23.3)	ns
Insulin (mU/L)	22.8 (12.9)	27.4 (16.3)	25.2 (14.9)	ns
HOMA‐IR	5.0 (3.0)	6.2 (3.6)	5.6 (3.4)	ns
Matsuda Index	2.4 (1.5)	2.1 (1.5)	2.2 (1.5)	ns
IGI	3.4 (2.3)	3.5 (2.3)	3.5 (2.3)	ns
oDI	6.3 (3.3)	5.7 (3.7)	6.0 (3.5)	ns
Zonulina(ng/ml)	50.2 (6.8)	50.6 (6.3)	50.4 (6.5)	ns
Ln LBP (ug/ml)	2.9 (0.3)	3.0 (0.3)	2.9 (0.3)	ns
Ln IL‐6 (pg/ml)	2.1 (3.6)	2.5 (4.4)	2.4 (4.1)	ns

*Notes*: Data expressed as number (percentage) or mean (SD). The Student *t*‐test and the Pearson's chi‐squared test for categorical variables were used to test the differences according to sex. Ns. non‐significant M vs F.

Abbreviations: ALT, alanine aminotransferase; BMI, body mass index; HbA1c, glycated haemoglobin; HDL‐c, high‐density lipoprotein cholesterol; HOMA‐IR, Homeostatic Model Assessment‐insulin resistance; IGI, Insulinogenic Index; IL‐6, Interleukin‐6; LBP, lipopolysaccharide‐binding protein; LDL‐c, low‐density lipoprotein cholesterol; NAFLD, non‐alcoholic fatty liver disease; oDI, Oral Disposition Index; PAD, diastolic blood pressure; PAS, systolic blood pressure; SDS, standard deviation score; TC, total cholesterol; TG, triglyceride.

**TABLE 2:** Linear regression models. Dependent variable: Oral Disposition Index

Dependent variable	Independent variables	β	95% CIs		*p*
*Oral Disposition Index* ^*1*^	Constant		10.482	23.845	0.000
	Sex (1 = F)	−0.008	−1.456	1.343	0.936
	Age (years)	−0.307	−0.769	−0.152	**0.004**
	BMI SDS	0.003	−0.737	0.756	0.980
	Zonulin	−0.243	−0.240	−0.022	**0.019**
*Oral Disposition Index* ^*2*^	Constant		12.936	28.181	0.000
	Sex (1 = F)	−0.057	−2.176	1.394	0.663
	Age (years)	−0.516	−1.205	−0.199	**0.007**
	BMI SDS	0.041	−0.755	1.060	0.738
	Pubertal stage	0.229	−0.812	3.086	0.248
	Zonulin	−0.262	−0.270	−0.018	**0.026**

*Note*: Model^1^: R^2^ = 0.16; *p* = 0.005; Model^2^ (*n* = 71): *R* ^2^ = 0.214; *p* = 0.008. Bold indicates significant *p*‐values.

Abbreviation: BMI‐SDS, body mass index–standard deviation score.

## 4 DISCUSSION

The results of this study show that, among children and adolescents with overweight and obesity, having an increased intestinal permeability estimated by zonulin assay, was associated with a decreased oDI, independent of sex, and BMI SDS. The oDI has been proposed as a reliable predictor of T2DM progression: it reflects the inability of pancreatic beta cells to compensate for insulin resistance, being considered a predictor of conversion to disease as its value decreases.^9^

Altered intestinal permeability is not easy to assess. The serum zonulin assay has been considered as an easy to perform surrogate marker of dysfunctional intestinal barrier, suitable also in paediatric age.^10^, ^11^ Zonulin is a protein that reversibly controls intestinal permeability by regulating the binding between the epithelial cells of the intestinal mucosa.^4^ Intestinal dysbiosis, characteristic of subjects who consume a high‐fat and low‐fibre diet,^12^ stimulates the production of zonulin by the lamina propria of the intestinal epithelium.^4^ Zonulin secretion has been shown to be MyD88‐dependent and is followed by an increase in gut permeability secondary to the disassembly of the zonula occludens‐1 from the tight junctional complex.^4^ Activation of the zonulin pathway may represent a defensive mechanism that “flushes out” microorganisms, contributing to the host's innate immune response against changes in the microbiome ecosystem (small intestinal bacterial growth and/or dysbiosis).^5^ The increased intestinal permeability leads to the translocation of bacterial substances, as LPS, and products of their metabolism, as short‐chain fatty acids, in the systemic circulation, eventually determining a low‐grade inflammatory status, also called endotoxemia that leads to metabolic dysregulation.^3^

The association between altered intestinal permeability and impaired glucose metabolism was reported by Vangipurapu et al. who found an association between several microbial metabolites related to the gut microbiota, passed into circulation due to leaks in the intestinal barrier, with an increased risk of incident type 2 diabetes in adults.^13^ Accordingly, a negative correlation between circulating indolepropionic Acid, a microbial metabolite of tryptophan, and the onset of T2DM was found in a 1‐year follow‐up of subjects with impaired glucose tolerance.^14^ Pacifico et al. showed a positive association between serum zonulin and NAFLD, and a negative association between zonulin and whole‐body insulin sensitivity index.^15^ Similarly, Kume et al. and Kim et al. showed a positive correlation between serum zonulin and levels of insulin resistance and BMI SDS in groups of children and adolescents with obesity.^16^, ^17^

The new contribution of our study consists in demonstrating for the first time an association between zonulin and oDI, that is, a risk factor for the development of T2DM, independent of the level of obesity. Therefore, zonulin could be proposed not just as a marker of altered intestinal permeability in children and adolescents with overweight and obesity but also as a possible indicator of metabolism dysregulation helping to identify patients at increased risk of developing T2DM worthy of early and targeted preventive and therapeutic interventions.

Moreover, our findings sustain the central role of dysbiosis and intestinal permeability in early glucose metabolism dysregulation of obesity. The control of dysbiosis may contribute to improve glucose metabolism in obesity. Changes in dietary habits consisting of a reduction in fat and an increase in fibre intake induce a significant decrease in intestinal permeability,^18^ also independent of a decrease in BMI.^19^ Accordingly, in a cross‐sectional study, the use of inulin‐enriched pasta induced a reduction in circulating zonulin levels.^19^ Finally, in addition to diet, intake of prebiotics or probiotics could also improve dysbiosis and consequently reduce intestinal permeability in patients with obesity.^20^

Our study has some limitations: i) the sample size is quite small; ii) the cross‐sectional design does not allow to explore causality; iii) unavailability of data on diet that has an important influence on the microbiota and consequently on intestinal permeability; iv) the absence of measures of adiposity to be included in the model and v) the absence of normal‐weight subjects that could further clarify the independence of the relationship between zonulin and oDI from BMI SDS.

To conclude, our study is the first to demonstrate that, among children and adolescents with overweight and obesity, having increased serum zonulin levels (i.e. a plausible marker of altered intestinal permeability) is associated with an increased risk of potential progression to T2DM, independent of sex and BMI SDS.

## CONFLICT OF INTEREST

The authors have no conflicts of interest to declare.
